# Endoscopist’s Satisfaction with the Insertion Phase of Colonoscopy Is a Potential Quality Indicator for Colorectal Polyp Detection: A Propensity Score Matching Study

**DOI:** 10.5152/tjg.2024.23508

**Published:** 2024-06-01

**Authors:** Fumiaki Ishibashi, Sho Suzuki, Kentaro Mochida, Mizuki Nagai, Konomi Kobayashi, Tomohiro Kawakami, Tetsuo Morishita

**Affiliations:** 1 Department of Gastroenterology, International University of Health and Welfare Ichikawa Hospital, Chiba, Japan; 2 Koganei Tsurukame Clinic, Endoscopy Center, Tokyo, Japan

**Keywords:** Colonoscopy, quality indicator, insertion time, polyp detection rate, adenoma detection rate

## Abstract

**Background/Aims:**

Quality indicators during the insertion phase of colonoscopy require exploration. Unsatisfactory insertion experiences cause endoscopist psychophysiological fatigue and affect the quality of their inspection. This comparative study used propensity score matching (PSM) to determine whether endoscopist satisfaction during scope insertion was related to polyp detection rate (PDR).

**Materials and Methods:**

Patients who underwent colonoscopy screening between April 2019 and December 2022 were enrolled in this study. The endoscopist satisfaction score (high and low) during the insertion phase in each examination was recorded based on the level of fatigue and presence of paradoxical scope movement. All examinations were classified into 2 groups: a high and a low satisfaction score group. After PSM with potential confounding factors related to polyp detection (endoscopist, insertion and withdrawal time, and sedative agent use), the PDR and adenoma detection rate (ADR) were compared.

**Results:**

Overall, 4142 patients (average age, 54.1 years old; 54.4% male) underwent colonoscopies performed by twelve experienced endoscopists. Analysis using a logistic regression model revealed that a high satisfaction score during the insertion phase was an independent predictor of polyp detection (*P* < .001, odds ratio 1.79, 95% CI 1.41-2.33), whereas insertion time was not. After PSM, 513 patients from both groups were eligible for comparison. Polyp detection rate and ADR were significantly higher in the high-satisfaction group than in the low-satisfaction group (49.5% vs. 36.6%, *P* < .001; 35.1% vs. 27.1%, *P* = .007).

**Conclusion:**

The endoscopists’ level of satisfaction with the insertion phase was shown to be a potential predictor of PDR in screening colonoscopy.

**Main Points**

- Quality indicators that predict individual polyp detection during the insertion phase of colonoscopy are useful in daily practice.
- Unsatisfactory scope insertion experience potentially affects the quality of the endoscopist’s inspection during the withdrawal phase, however, it has been difficult to quantify.
- The endoscopist satisfaction score, defined by the level of fatigue and presence of paradoxical scope movement at the time of insertion into the cecum, was closely related to the polyp detection rate.

## Introduction

Colorectal cancer is the second leading cause of cancer death in Japan and the United States; thus, screening strategies to further reduce deaths from colorectal cancer are crucial.^1,2^ The adenoma detection rate (ADR) was first reported as a quality indicator in screening colonoscopy, with a reduction in colorectal cancer death as the primary endpoint.^3,4^ However, it is difficult to evaluate the ADR based solely on screening colonoscopies because pathological diagnosis is required to calculate the ADR. In this context, other useful indicators in clinical settings have been explored. For example, the polyp detection rate (PDR), which does not require pathological diagnosis, has been proven to be a useful alternative indicator. In addition, several studies have investigated whether withdrawal time and the degree of bowel preparation can predict polyp detection.^5-7^ As part of this line of research, scope insertion time has also been investigated as a possible predictor of polyp detection; however, conflicting reports have failed to provide a consensus. While some reports suggest that longer insertion times lead to lower ADRs,^8^ there are also reports that indicate that the ADR does not decrease as long as the withdrawal time is ensured, even if the insertion time is long.^9^ Relatedly, we have also reported that endoscopists can improve their ADRs by providing them with individual feedback on performance, particularly on withdrawal time and ADR, followed by real-time monitoring of withdrawal time, regardless of the length of the insertion time.^10^ Insertion time, by itself, is not well-established as a quality indicator because it is greatly affected by the endoscopist’s skill, difficulty of scope insertion, and use of sedative agents.

Endoscopic insertion difficulty is often quantified by insertion time.^11^ However, in the practice of minimal sedation colonoscopy, scope insertions are performed with care, even if the insertion time takes longer, to lessen pain and to limit the amount of sedative agent used.^9^ In this study, we hypothesized that even if insertion takes longer or requires more sedative agents, the quality of the examination at the time of withdrawal may be assured if the endoscopist is satisfied with the insertion. The aim of this study is to determine whether endoscopist satisfaction during the insertion phase could be a quality indicator in minimal sedation colonoscopy.

## Materials and Methods

### Study Design

This was a multi-center, retrospective, comparative study using propensity score matching (PSM). The study design was approved by the Institutional Review Board of Shinjuku Tsurukame Clinic on January 11, 2023 (approval number: 2201). Written informed consent was not obtained from all participants. Instead, the study plan was publicized by posting the study protocol in the clinic and on the website, and patients who did not wish to participate in the study were excluded through an opt-out option. This study was conducted according to the guidelines of the Declaration of Helsinki.

### Subjects

The medical data of asymptomatic patients aged between 20 and 80 years who underwent screening colonoscopies at Koganei Tsurukame Clinic and International University of Health and Welfare Ichikawa Hospital from April 2019 to March 2023 were retrospectively reviewed. Patients with advanced colorectal cancer, inflammatory bowel disease, familial colorectal adenomatosis, or a history of surgical colorectal resection were excluded.

As a bowel preparation protocol, the method validated in a previous study was used; a senna laxative (Alozen® 1 g/day) or polyethylene glycol (PEG) (Movicol® 120 mL/day) was administered for 3 days prior to the examination, followed by 2000 mL of PEG-electrolyte lavage solution (PEG-ELS, Niflec®) on the day of the examination.^12^ If 2000 mL of PEG-ELS was insufficient to achieve cleansing, another 300 mL of PEG-ELS was administered intermittently. If this was still insufficient, a high-pressure enema was administered. All patients underwent colonoscopy immediately after finishing bowel preparation in the morning.

### Sedation Method

Moderate sedation was used in this study.^13^ All patients were instructed not to use sedative agents at the beginning of the examination unless they experienced pain during previous endoscopic examinations. If sedative agents were desired, low-dose propofol was used unless there were contraindications. Sedation with a combination of pethidine hydrochloride and midazolam was only employed when propofol could not be used owing to allergies or contraindications. In this study, the appropriate level of sedation was 2-4 on the Modified Observer’s Assessment of Alertness/Sedation Scale. An initial bolus of propofol at 1 mg/kg was administered. If the desired level of sedation was not reached, propofol was added at 10-20 mg increments every minute. Patients in whom propofol use was contraindicated received a bolus dose of 35 mg of pethidine hydrochloride. When midazolam was used, a bolus dose of 2 mg was administered, followed by an additional dose of 1 mg every 2 minutes if the desired sedation level was not reached. Oxygen saturation, blood pressure, and pulse rate were continuously monitored during sedation, and at least 2 paramedics and 1 nurse, in addition to the endoscopist, constantly observed the patient’s condition.

### Training of the Endoscopists

The endoscopists participating in this study were trained for at least 2 years on the scope insertion technique for access through the sigmoid colon using the Endoscope Position Detecting Unit (UPD) (Olympus, Tokyo, Japan).^14,15^ All endoscopists were tested using UPD to see if they had achieved their ideal insertion technique, including testing on a pattern that passes through the sigmoid colon without forming a loop or a pattern that forms a loop but releases midway and reaches the cecum in a straight position. All endoscopists were certified by the Japanese Society of Gastrointestinal Endoscopy for the completion of the endoscopy training course.

### Procedures

For scope insertion, a transparent attachment was used as it protruded 1 mm from the tip of the endoscope. The water-exchange method was not used. CO_2_ insufflation was performed during the examination. During insertion, the endoscopist tried to use the least painful insertion method; however, if pain occurred, minimal sedation was performed.

Endoscopist fatigue level was immediately evaluated based on the numerical rating scale (0-10) at the time of cecum intubation. A score of 6 or higher was considered “high” fatigue. Endoscopists were instructed to include both physical and mental fatigue. Endoscopist satisfaction score (high or low) was eventually decided by the fatigue level and whether the endoscope screen moved paradoxically when the scope was pushed or pulled (Table 1). Endoscopist satisfaction score was recorded by the endoscopy room nurse at the time of cecum intubation and finally appeared in colonoscopy reports. In our institutions, the satisfaction score is routinely recorded for each examination.

Based on the results of a previous study,^10^ the endoscopists were instructed to spend more than 6 minutes for observation during the withdrawal phase, and real-time monitoring of the observation time was performed in all examinations to ensure an adequate ADR. At the time of observation, the endoscopist scored each segment of the colon (right, transverse, and left colon) for the extent of bowel preparation according to the Boston Bowel Preparation Scale (BBPS),^16^ which was noted after the examination.

All identified polyps were recorded after the completion of the examination. At the time of polyp identification, a magnified observation with narrow band imaging was performed to make a differential diagnosis between adenomatous polyps, sessile serrated lesions (SSLs), and non-neoplastic polyps. When adenomatous polyps or SSLs were diagnosed, a polypectomy was performed.

In this study, the endoscope models used were CF-HQ290Z or PCF-H290Z (Olympus, Tokyo, Japan). CF-HQ290Z was recommended for use on obese patients with a body mass index ≥ 25. The light source used was an EVIS-LUCERA ELITE system (Olympus, Tokyo, Japan).

### Statistical Analysis

Patients who met the inclusion criteria were selected from the database during the study period. Subsequently, age, sex, the endoscopist’s name, endoscopist’s satisfaction during insertion, insertion time, withdrawal time, BBPS, presence of a diverticulum in the sigmoid colon, sedative agent use, presence of polyp resection, and pathological diagnosis of resected polyps were all investigated.

First, all patients were divided into groups according to the presence or absence of polyps, and their backgrounds were compared using univariate analysis. The predictors of polyp detection were analyzed using a logistic regression model for explanatory variables that showed significant differences in the univariate analysis.

Next, all patients were divided into a high satisfaction score group and a low satisfaction score group according to endoscopist satisfaction during insertion. To analyze the predictive factors for high endoscopist satisfaction, a logistic regression model was used, with patient background as an explanatory variable. The stepwise method was used to determine the combination of explanatory variables that would take the smallest Akaike’s Information Criterion. Propensity score matching was performed to match the case backgrounds of the 2 groups to combinations of explanatory variables. Propensity score matching used the nearest neighbor method with 1 : 1 matching. According to the pathological diagnosis of colorectal polyps, the PDR, ADR, SSL detection rate (SDR), and advanced ADR (AADR) were calculated. Advanced adenomas were defined as high-grade adenomas, adenomas larger than 10 mm, or villous adenomas.

All statistical analyses were performed using R version 4.0.4.^17^ In univariate analysis, the chi-square test was applied for categorical variables, and Student’s *t*-test was applied for continuous variables. The logistic regression model was run using the glm function of the R software, and the step function was used to select explanatory variables using the stepwise method. The MatchIt package was used for PSM. Data for continuous variables were expressed as mean ± standard deviation. Statistical significance was set at *P* < .05.

## Results

### Baseline Characteristics of Patients and Endoscopists

During the study period, 4308 patients underwent colonoscopies conducted by 12 endoscopists. After excluding 23 patients with advanced cancer, 112 patients with inflammatory bowel disease, and 31 post-colorectal surgery patients, 4142 patients were eligible for the analysis. The endoscopic performances of the 12 endoscopists are shown in Table 2. Among all endoscopists, over 60% of the examinations performed resulted in high levels of satisfaction with the scope insertions. The insertion time varied among endoscopists, but no relationship was found between insertion time and polyp detection (Table 2).

### Comparison of Groups with and without Polyp Detection

**Figure 1.**
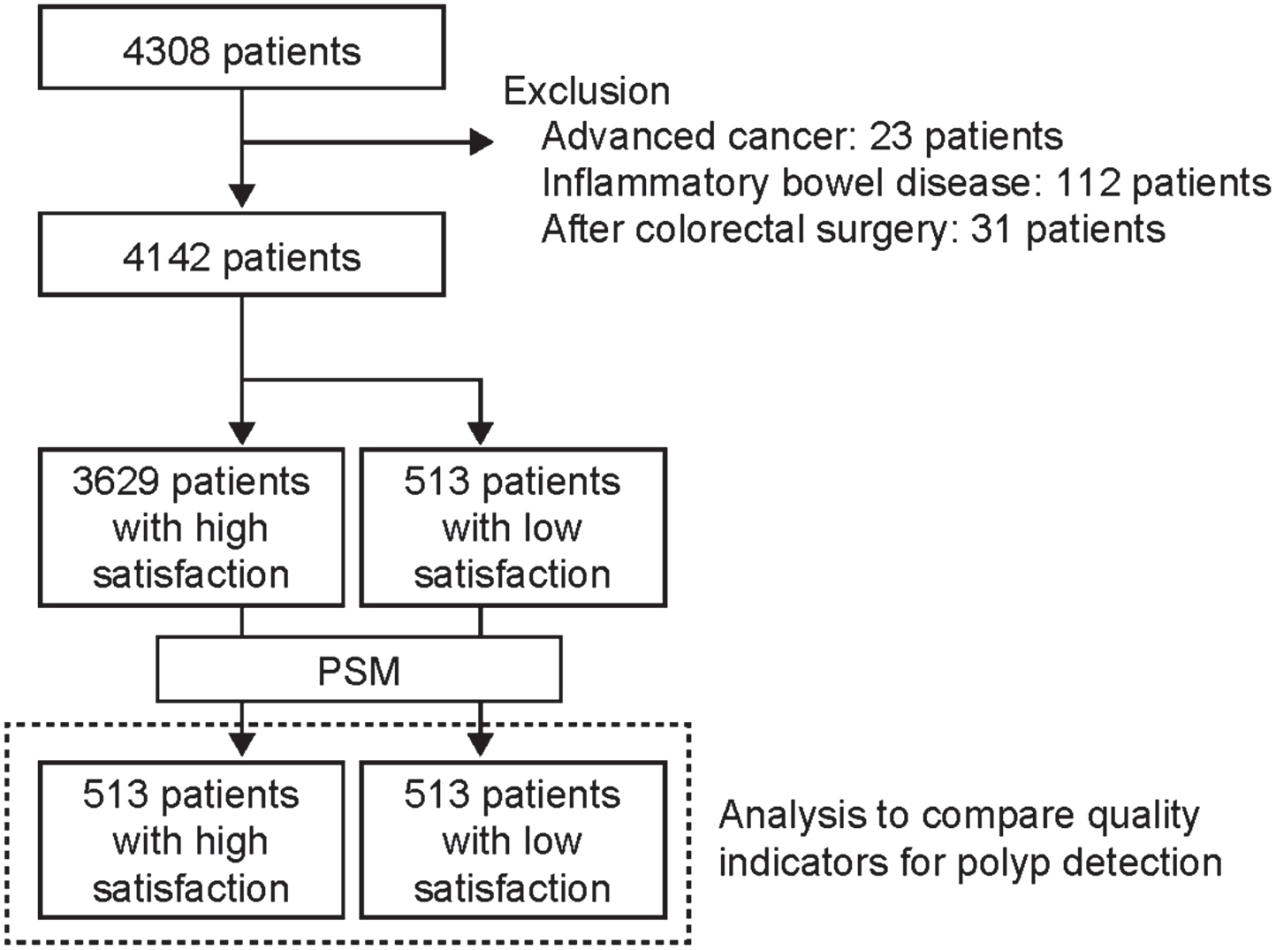
Study flow diagram. A total of 4308 patients were initially enrolled in the study. Subsequently, 86 patients were excluded, and as a result, 4142 patients were ultimately eligible for the study. Of these patients, 3629 were classified into the high satisfaction group. The remaining 513 patients were classified into the low satisfaction group. After propensity score matching (PSM), 513 patients were compared for quality indicators of polyp detection.

The 4142 patients were classified into 2 groups according to the presence or absence of polyps as follows: 2073 patients were included in the group with polyps and 2069 patients comprised the group without polyps (Figure 1). Univariate analysis of patient backgrounds between the 2 groups showed that patients in the group with polyps were significantly older (57.6 vs. 50.6 years old, *P* < .001), included more males (71.0% vs. 47.4%, *P* < .001), had a higher percentage of endoscopist A examinations (46.9% vs. 34.3%, *P* < .001), had more insertions with high satisfaction levels (90.9% vs. 84.1%, *P* < .001), shorter insertion time (4.7 vs. 5.4 min, *P* < .001), had a longer withdrawal time (10.1 vs. 6.4 min, *P* < .001), and showed lower use of sedative agents (46.3% vs. 52.7%, *P* < .001) (Table 3). There was no difference in the degree of bowel cleansing (BBPS) or the presence of diverticula between the 2 groups (Table 3). Patient background with significant differences was used as an explanatory variable, and predictors of polyp identification were analyzed using a logistic regression model. The results showed that older age (*P* < .001, odds ratio (OR) 2.53, 95% confidence interval (CI) 2.18-2.93), male sex (*P* < .001, OR 1.86, 95% CI 1.60-2.16), insertion with a high satisfaction level (*P* < .001, OR 1.79, 95% CI 1.41-2.33), and longer withdrawal time (*P* < .001, OR 17.6, 13.1-23.5) were independent predictors (Table 3). In addition, differences among endoscopists were associated with polyp detection.

### Comparison of Groups with High and Low Endoscopist’s Satisfaction During Insertion

Next, all patients were reclassified according to the endoscopist’s satisfaction level and background factors were compared. As a result, there was a large difference in the number of patients between the 2 groups (3629 patients in the high satisfaction group vs. 513 patients in the low satisfaction group). Differences between the 2 groups were observed with respect to background factors, such as the percentage of males (56.9% vs. 36.8%), insertion time (4.4 vs. 9.6 minutes), and sedative agent use (47.6% vs. 63.4%). Therefore, a simple comparison of quality indicators for polyp detection was not possible (Table 4). After matching the patient backgrounds using PSM to validate the comparison between the 2 groups, the absolute standardized differences in the background factors decreased (Table 4). A comparison of predictors for polyp detection among the matched patients showed that the PDR (49.5% vs. 36.6%, *P* < .001) and ADR (35.1% vs. 27.1%, *P* = .007) were significantly higher in the high-satisfaction group (Table 5).

## Discussion

Many quality indicators in colonoscopy, such as ADR, PDR, withdrawal time, and degree of bowel preparation, can only be quantified after the examination is completed. An indicator that can predict the quality of the examination during the test is thus vital, as it enables the endoscopist to actively modify procedural outcomes, such as polyp detection, even within the test. In this study, we explored a new quality indicator that can be used during the insertion phase of a colonoscopy. PSM was implemented to adjust for all confounding factors in the insertion phase, such as scope insertion time, sedative agent use, and endoscopist skill. Of note, patient’s background including age, sex, and body mass index was also adjusted by PSM. The previous report showed that insertion time could be related to difficulty in intubating the cecum and low body mass index. Female and older age were risk factors for loner intubation time.^18^ In this study, PSM effectively homogenized these potential confounding factors between the low and high satisfaction groups. In addition, after PSM, there was no difference between the groups in withdrawal time, which is known as a quality indicator for colonoscopy. The results showed that endoscopist satisfaction during scope insertion could be considered as an alternative new quality indicator encompassing all factors in the insertion phase.

Whether endoscopists’ fatigue levels affect the quality of examinations has been debated in recent years. In fact, it has been shown that when multiple colonoscopies are performed on the same day, the quality of the exam decreases as the frequency of examinations increases.^19,20^ Similarly, it has been reported that ADR improves when colonoscopy is performed in the morning rather than in the afternoon.^21^ In contrast, some reports suggest that delays in the timing of examination and the number of examinations performed are not associated with ADR.^22,23^ None of these reports directly measured endoscopist fatigue or investigated its relationship with examination quality. This study attempted to settle this debate by using endoscopist satisfaction during insertion as a surrogate measure of fatigue. Our results suggest that even if scope insertion takes longer, the quality of observation during the withdrawal phase can be maintained at a high degree if the insertion method was satisfactory to the endoscopist and caused less mental and physical fatigue.

Several studies have investigated whether the insertion time can be a quality indicator in the insertion phase. However, it was suggested that this may not be a quality indicator because the insertion time is greatly affected by the skill of the endoscopist, insertion method (e.g., whether the water exchange method is used), and use of sedatives. In fact, in this study, the analysis of performance by endoscopists failed to reveal a trend of higher ADRs with shorter insertion times (Table 3). Furthermore, the results of a logistic regression model analysis of all cases with the presence or absence of polyps showed that differences in the endoscopist, insertion pattern, and use of sedative agents were predictive factors for polyp identification, but insertion time was not. A shorter insertion time was expected to reduce the total examination time, and thus the amount of sedation and patient discomfort, but its contribution to polyp detection was found to be negligible.

This study has some limitations owing to the nature of the study design. Selection bias cannot be ruled out, and the generalizability of the results may be low. Since endoscopist satisfaction during the insertion phase depends on the endoscopist’s subjective views, a randomized controlled trial (RCT) could not be designed. Instead, a pseudo-RCT design using PSM was utilized. In addition, all endoscopists enrolled in the study were specialists in colonoscopy (all had high satisfaction examination ratings >60%). Therefore, we cannot deny the possibility that the distribution of the high- and low-satisfaction groups included in this study may deviate from that in the general population. If this study was conducted by less experienced endoscopists, there is a possibility of low reproducibility. In the future, more endoscopists with diverse backgrounds will be required to validate whether endoscopist satisfaction during the insertion phase can be a quality indicator.

The results of the PSM-based comparative study showed that endoscopist satisfaction during scope insertion could be a potential quality indicator for polyp detection, which can be determined in the insertion phase of colonoscopy.

## Figures and Tables

**Table 1.** Definition of Endoscopist Satisfaction During Insertion Phase

		Endoscopist Fatigue Level	
		Weak (NRS 0-5)	Strong (NRS 6-10)
Scope paradoxical movement	Absent	High satisfaction	Low satisfaction
	Present	Low satisfaction	Low satisfaction

NRS, numerical rating scale.

**Table 2.** Endoscopist Characteristics and Examination Performance

	Endoscopist											
	A	B	C	D	E	F	G	H	I	J	K	L
Endoscopist characteristics												
Experience (years)	12	12	10	15	12	14	10	7	8	11	10	9
Certified member of JSGE	Yes	Yes	Yes	Yes	Yes	Yes	Yes	Yes	Yes	Yes	Yes	Yes
Number of colonoscopies	1682	467	207	106	174	117	107	68	79	639	362	55
Patient characteristics												
Age (years)	52.7 ± 12.5	52.3 ± 11.3	50.2 ± 11.0	53.1 ± 12.2	64.0 ± 15.2	67.3 ± 13.0	59.1 ± 14.3	63.0 ± 15.9	65.5 ± 14.6	53.5 ± 13.0	53.1 ± 11.6	50.6 ± 10.36 ± 10.3
Sex (male: female)	889: 793	260: 207	171: 115	51: 55	111: 64	68: 49	65: 42	39: 29	34: 45	333: 306	209: 153	24: 31
Body mass index	22.4 ± 1.2	21.8 ± 1.7	22.2 ± 1.9	22.5 ± 1.8	22.5 ± 1.6	22.3 ± 1.7	22.4 ± 2.0	22.4 ± 2.1	22.2 ± 1.9	22.4 ± 1.4	22.6 ± 1.4	22.1 ± 2.11 ± 2.1
Bowel preparation level (BBPS)	8.3 ± 1.3	8.3 ± 1.1	8.7 ± 0.8	8.1 ± 1.4	7.6 ± 1.9	7.8 ± 2.4	6.8 ± 2.1	6.8 ± 2.8	7.6 ± 1.5	8.4 ± 1.1	8.6 ± 0.8	8.7 ± 07 ± 0.7
Examination performance												
Satisfaction level (high satisfaction) (%)	1492 (88.7)	446 (95.5)	207 (72.4)	65 (61.3)	155 (89.1)	95 (81.2)	91 (85.0)	52 (76.5)	67 (84.8)	567 (88.7)	344 (95.0)	45 (81.8)
Insertion time (min)	4.7 ± 2.7	4.1 ± 2.4	8.5 ± 6.7	8.2 ± 4.5	4.7 ± 2.8	4.9 ± 3.4	6.2 ± 3.7	8.2 ± 4.6	6.5 ± 5.1	4.4 ± 2.6	4.5 ± 2.9	8.5 ± 4.75 ± 4.7
Withdrawal time (min)	8.4 ± 2.8	6.2 ± 3.4	8.9 ± 4.0	9.0 ± 6.2	10.4 ± 3.4	10.3 ± 6.1	8.1 ± 4.3	12.7 ± 6.7	13.7 ± 6.7	8.0 ± 2.6	6.9 ± 3.5	9.9 ± 3.89 ± 3.8
Sedative agent use (%)	811 (48.2)	222 (47.5)	122 (42.7)	39 (36.8)	89 (51.1)	49 (41.9)	44 (41.1)	35 (51.5)	39 (49.4)	358 (56.0)	207 (57.2)	36 (65.5)
Polyp detection (PDR)	972 (57.8)	137 (29.3)	90 (31.5)	39 (36.8)	121 (69.5)	80 (68.4)	54 (50.5)	41 (60.3)	41 (51.9)	364 (57.0)	112 (30.9)	22 (40.0)
Adenoma detection (ADR)	610 (36.3)	111 (23.8)	68 (23.8)	27 (25.4)	103 (59.2)	73 (62.4)	49 (45.8)	36 (52.9)	35 (44.3)	306 (47.9)	100 (27.6)	18 (32.7)
SSL detection (SDR)	53 (3.1)	3 (0.6)	2 (0.7)	0 (0)	15 (8.6)	10 (8.5)	2 (1.9)	4 (5.9)	1 (1.3)	72 (11.3)	6 (1.7)	0 (0)
Advanced adenoma detection (AADR)	64 (3.8)	11 (2.4)	9 (2.1)	3 (2.8)	36 (20.7)	23 (19.7)	10 (9.3)	14 (20.6)	10 (12.7)	22 (3.4)	10 (2.8)	2 (3.6)
Carcinoma detection rate	4 (0.2)	0 (0)	0 (0)	0 (0)	10 (5.7)	5 (4.3)	2 (1.9)	2 (2.9)	2 (2.5)	13 (2.0)	6 (1.7)	1 (1.8)

Age, insertion time, withdrawal time, and BBPS are expressed as the mean ± SD. AADR, advanced adenoma detection rate; ADR, adenoma detection rate; BBPS, Boston Bowel Preparation Scale; JSGE, Japanese Society of Gastrointestinal Endoscopy; PDR, polyp detection rate; SDR, serrated lesion detection rate; SSL, sessile serrated lesion.

**Table 3.** Comparison of Background Characteristics and Endoscopist’s Performance for Polyp Detection

	Polyp Detected (n = 2073)	Polyp Not Detected (n = 2069)	Univariate Analysis	Logistic Regression Analysis	
			*P*	OR (95% CI)	*P*
Age (years old)	57.6 ± 12.0	50.6 ± 136 ± 13.3	<.001		
≦50 years old (%)	601 (29.0)	1088 (52.6)		Reference	
>50 years old	1472 (71.0)	981 (47.4)		2.53 (2.18-2.93)	<.001
Sex			<.001		
Female (%)	769 (37.1)	1120 (54.1)		Reference	
Male (%)	1304 (62.9)	949 (45.9)		1.86 (1.60-2.16)	<.001
Body mass index			.238		
>22	1116 (53.8)	1075 (52.0)		Reference	
≦22	957 (46.2)	994 (48.0)		0.97 (0.92-1.12)	.321
Endoscopist			<.001		
Endoscopist A (%)	972 (46.9)	710 (34.3)		Reference	
Endoscopist B (%)	137 (6.6)	330 (15.9)		0.29 (0.23-0.37)	<.001
Endoscopist C (%)	90 (4.3)	196 (9.5)		0.24 (0.17-0.33)	<.001
Endoscopist D (%)	39 (1.9)	67 (3.2)		0.37 (0.22-0.60)	<.001
Endoscopist E (%)	121 (5.8)	53 (2.6)		0.95 (0.64-1.40)	.787
Endoscopist F (%)	80 (3.9)	37 (1.8)		1.08 (0.69-1.71)	.733
Endoscopist G (%)	54 (2.6)	53 (2.6)		0.60 (0.38-0.93)	.022
Endoscopist H (%)	41 (2.0)	27 (1.3)		0.38 (0.20-0.73)	.003
Endoscopist I (%)	41 (2.0)	38 (1.8)		0.16 (0.09-0.29)	<.001
Endoscopist J (%)	364 (17.6)	275 (13.3)		1.00 (0.82-1.23)	.967
Endoscopist K (%)	112 (5.4)	250 (12.1)		0.29 (0.22-0.38)	<.001
Endoscopist L (%)	22 (1.1)	33 (1.6)		0.27 (0.13-0.57)	<.001
Satisfaction level during insertion			<.001		
Low satisfaction (%)	188 (9.1)	329 (15.9)		Reference	
High satisfaction (%)	1885 (90.9)	1740 (84.1)		1.79 (1.41-2.33)	<.001
Insertion time (min)	4.7 ± 3.1	5.4 ± 44 ± 4.0	<.001		
>6 min	438 (21.1)	576 (27.8)		Reference	
≦6 min	1635 (78.9)	1493 (72.2)		1.13 (0.94-1.39)	.189
Withdrawal time (min)	10.1 ± 4.4	6.4 ± 14 ± 1.8	<.001		
≦10 min	1365 (65.8)	2001 (96.7)		Reference	
>10 min	708 (34.2)	68 (3.3)		17.6 (13.1-23.5)	<.001
Bowel preparation level (BBPS)			.054		
≦6	254 (12.3)	205 (9.9)		Reference	
>6	1819 (87.7)	1864 (90.1)		1.11 (0.88-1.41)	.369
Sedative agent use (%)	959 (46.3)	1092 (52.7)	<.001	0.98 (0.85-1.13)	.777

Age, insertion time, withdrawal time, and BBPS are expressed as the mean ± SD.

BBPS, Boston bowel preparation scale; OR, odds ratio.

**Table 4.** Patients and Endoscopists’ Background Before and After Matching

	Before PSM			After PSM		
	High Satisfaction (n = 3629)	Low Satisfaction (n = 513)	ASD	High Satisfaction (n = 513)	Low Satisfaction (n = 513)	ASD
Age (years)	53.9 ± 13.0	55.6 ± 14.2	0.125	55.7 ± 14.2	55.6 ± 14.2	0.007
Sex						
Male (%)	2065 (56.9)	189 (36.8)	0.411	175 (34.1)	189 (36.8)	0.056
Female (%)	1564 (43.1)	324 (63.2)	0.411	338 (65.9)	324 (63.2)	0.056
Body mass index	22.4 ± 1.4	22.1 ± 1.3	0.222	22.2 ± 1.3	22.1 ± 1.3	0.077
Endoscopist						
Endoscopist A (%)	1492 (41.1)	190 (37.0)	0.084	207 (40.4)	190 (37.0)	0.070
Endoscopist B (%)	446 (12.3)	21 (4.1)	0.302	23 (4.5)	21 (4.1)	0.020
Endoscopist C (%)	207 (5.7)	79 (15.4)	0.320	53 (10.3)	79 (15.4)	0.153
Endoscopist D (%)	65 (1.8)	41 (8.0)	0.290	36 (7.0)	41 (8.0)	0.038
Endoscopist E (%)	155 (4.3)	19 (3.7)	0.031	23 (4.5)	19 (3.7)	0.040
Endoscopist F (%)	95 (2.6)	22 (4.3)	0.093	25 (4.9)	22 (4.3)	0.029
Endoscopist G (%)	91 (2.6)	16 (3.1)	0.030	22 (4.3)	16 (3.1)	0.064
Endoscopist H (%)	52 (1.4)	16 (3.1)	0.115	13 (2.5)	16 (3.1)	0.036
Endoscopist I (%)	67 (1.8)	12 (2.3)	0.035	14 (2.7)	12 (2.3)	0.026
Endoscopist J (%)	569 (15.7)	70 (13.6)	0.059	78 (15.2)	70 (13.6)	0.046
Endoscopist K (%)	345 (9.5)	17 (3.3)	0.255	10 (1.9)	17 (3.3)	0.088
Endoscopist L (%)	45 (1.2)	10 (1.9)	0.057	9 (1.8)	10 (1.9)	0.007
Insertion time (min)	4.4 ± 2.7	9.6 ± 5.6	1.183	8.3 ± 4.0	9.6 ± 5.6	0.267
Withdrawal time (min)	8.3 ± 3.7	8.2 ± 4.4	0.025	8.3 ± 3.9	8.2 ± 4.4	0.024
Bowel preparation level (BBPS)	8.3 ± 1.3	8.1 ± 1.8	0.127	8.1 ± 1.6	8.1 ± 11 ± 1.8	0
Sedative agent use (%)	1726 (47.6)	325 (63.4)	0.322	332 (64.7)	325 (63.4)	0.027

Age, insertion time, withdrawal time, and BBPS are expressed as mean ± SD.

ASD, absolute standardized difference; BBPS, Boston bowel preparation scale; PSM, propensity score matching.

**Table 5.** Comparison of Quality Indicators for Polyp Detection After Matching

	High Satisfaction (n = 513)	Low Satisfaction (n = 513)	*P*
Polyp detection (PDR) (%)	254 (49.5)	188 (36.6)	<.001
Adenoma detection (ADR) (%)	180 (35.1)	139 (27.1)	.007
Sessile serrated lesion detection (SDR) (%)	19 (3.7)	13 (2.5)	.369
Advanced adenoma detection (AADR) (%)	30 (5.8)	19 (3.7)	.143

AADR, advanced adenoma detection rate; ADR, adenoma detection rate; PDR, polyp detection rate; SDR, sessile serrated lesion detection rate.

## Data Availability

All data generated or analyzed during this study are included in this article. Further inquiries can be directed to the corresponding author.
